# Exploring research trends and hotspots in PI3K/Akt signaling pathway in ischemic stroke: a bibliometric analysis

**DOI:** 10.3389/fnmol.2025.1613702

**Published:** 2025-06-23

**Authors:** Yingquan Liu, Yu Ye, Lin Bai, Fan Dai, Xingxing Su, Peijia Hu, Hongliang Cheng

**Affiliations:** ^1^The First Clinical College of Anhui University of Chinese Medicine, Hefei, China; ^2^Graduate School Anhui University of Chinese Medicine, Hefei, China; ^3^The Second Affiliated Hospital of Anhui University of Chinese Medicine, Hefei, China

**Keywords:** PI3K/AKT, signaling pathways, bibliometric analysis, ischemic stroke, visual analysis, scientific networks

## Abstract

**Objective:**

This study explores potential therapeutic strategies by determining the current research status, hotspots, and development trends through bibliometric analysis of the PI3K/Akt in ischemic stroke (IS).

**Methods:**

We searched the Web of Science Core Collection for publications on IS and the PI3K/Akt pathway, covering January 1, 2010, to December 31, 2024. VOSviewer and CiteSpace software were used to analyze research hotspots and cutting-edge topics in the field and generate visual maps of relevant countries, institutions, authors, journals, keywords, and references.

**Results:**

A total of 635 publications were analyzed. The number of publications indicates a steady annual increase in research output. China, Capital Medical University, and Wang Lei were identified as the most prolific country, institution, and author, respectively. The top three contributing journals were *Brain Research*, *Journal of Ethnopharmacology*, and *Frontiers in Pharmacology*. Autophagy, microglia and neuroinflammation, bioinformatics approaches, and traditional Chinese medicine (TCM) are not only current research areas but also important trends for future research. Notably, targeting IS with TCM holds significant potential for translating basic research findings into clinical applications.

**Conclusion:**

This bibliometric analysis provides an in-depth overview of research on the PI3K/Akt pathway in IS, revealing current research status, hotspots, and future research trends. This will provide valuable guidance and direction for developing novel therapeutic strategies targeting this pathway.

## 1 Introduction

Stroke is the second leading cause of death worldwide, and its incidence is closely related to the level of economic development. In low-income countries, about 20% of the population may be affected by stroke, while in high-income countries, this proportion is higher than 30% (Hilkens et al., 2024). More than 75% of stroke cases are ischemic stroke (IS) caused by arterial blockages (Xu et al., 2020)(Xu et al., 2020). IS is caused by three major vascular factors: large artery disease, cardiogenic embolism, and small vessel disease (Hilkens et al., 2024). Inadequate or disrupted cerebral blood supply triggers a cascade of signal transduction events, leading to irreversible brain damage, neurological deficits, or even death (Liu et al., 2025). Clinically, pharmacological thrombolysis is the primary treatment for IS. However, its therapeutic window is only about 4 h, and the high risk of bleeding significantly limits its effectiveness (Wu et al., 2020). Therefore, novel therapeutic approaches to prevent and/or treat IS are urgently needed.

Phosphoinositide 3-kinase (PI3K) is a class of plasma membrane-associated lipid kinases comprising two regulatory subunits (p38 and p55) and a catalytic subunit (p110) (Yang et al., 2019). Phosphoinositide 3-kinase induces conformational changes in protein kinase B (Akt), leading to its phosphorylation, activation, and binding to the cellular membrane (Xie Y. et al., 2018). The PI3K/Akt signaling pathway (hereinafter referred to as PI3K/Akt) is an essential intracellular regulator of cell proliferation, differentiation, chemotaxis, survival, metabolism, and angiogenesis (Katso et al., 2001; Engelman et al., 2006). The pathogenesis of IS is complex, involving inflammatory responses triggered by cerebral ischemia, toxic effects of excitatory amino acids, oxidative stress, autophagy, apoptosis, and vascular endothelial stability (Liu T. et al., 2023). The PI3K/Akt is significantly involved in the pathological mechanisms of IS and, therefore, has become the focus of recent research (Xie Y. et al., 2018; Zheng et al., 2024). However, the current understanding of the PI3K/Akt in IS is still in its early stage. Therefore, an in-depth study of its role in IS holds significant promise.

Bibliometrics is a widely used statistical method for analyzing knowledge accumulation and research trends in a given field (Chen and Song, 2019). Recently, bibliometrics has been applied to IS research (Chen et al., 2023). However, a comprehensive bibliometric analysis of IS’s PI3K/Akt remains lacking. This study’s systematic bibliometric analysis of literature on the PI3K/Akt in IS was performed to assess the current status and global research trends. The findings will enhance researchers’ understanding of the PI3K/Akt and contribute to identifying novel strategies for IS prevention and treatment.

## 2 Materials and methods

This study did not involve animal experiments or human testing, so ethical review was not required.

### 2.1 Data collection and standardization

This study was searched based on the Web of Science Core Collection (WoSCC). The database features a strict journal selection mechanism and a standardized citation data system, which effectively ensures the homogeneity of bibliometric indicators and makes it the preferred choice for bibliometric research (Zhang et al., 2021; Yang J. et al., 2022; Veiga-del-Baño et al., 2023). Due to some delay in database inclusion, this may lead to systematic bias. To ensure that updates to the database have stabilized, a certain amount of time needs to be allowed for the deadline (December 31, 2024) set by this study for literature inclusion (Lv et al., 2025). Therefore, publications on PI3K/Akt in IS were searched and screened in the WoSCC database on February 1, 2025, for this study. Also excluded due to the small number of publications in this area before January 1, 2010. The search formula used was as follows: TS = (“PI3K/AKT” OR “PI3K/Akt” OR “PI3K-Akt” OR “Phosphoinositide-3 Kinase/Akt” OR “Phosphatidylinositol 3-Kinase/Akt” OR “Phosphoinositide-3 Kinase/protein kinase B” OR “Phosphatidylinositol 3-Kinase/protein kinase B” OR “Phosphoinositide-3 Kinase/serine/threonine kinase” OR “Phosphatidylinositol 3-Kinase/serine/threonine kinase” OR “Phosphatidylinositol 3-Kinase/serine/threonine protein kinase” OR “Phosphatidylinositol-3-hydroxykinase/protein kinase B”) AND TS = (“ischemic stroke” OR “Ischemic Strokes” OR “Stroke, Ischemic” OR “Acute Ischemic Stroke” OR “Acute Ischemic Strokes” OR “cryptogenic ischemic stroke” OR “cryptogenic stroke” OR “cryptogenic embolism stroke” OR “wake up stroke” OR “wake-up stroke” OR “brain ischemia” OR “cerebral ischemia” OR “cerebral stem ischemia”). Based on the given timeframe and search terms, 958 relevant publications on the PI3K/Akt in IS were retrieved from the WOSCC database. Subsequently, we screened the 958 publications based on language type, publication type, and relevance to the search topic. Finally, 635 publications were identified for inclusion in this study’s bibliometric analysis. The complete records of the retrieved publications were exported in “txt” format for subsequent analyses.

In this study, the names of countries/regions and keywords were identified through VOSviewer software. The data were cleaned up using the merge and modify functions of the “thesaurus file.” For the names of countries/regions: (1) Replace the names of countries with internationally recognized names, e.g., replace “Peoples R China” with “China.” (2) The names of regions should be harmonized with the name of the country to which they belong, e.g., “Scotland” and “England” should be unified as “United Kingdom.” For keywords: (1) Combine synonyms according to the MeSH Thesaurus, e.g., “acute ischemic stroke” and “cerebral ischemic stroke” into “ischemic stroke.” (2) Merge of keywords with the same meaning but different forms of expression, e.g., “PI3K/Akt signaling” and “PI3K/Akt pathway” to PI3K/Akt; (3) The full name of the keyword is changed to an academically recognized abbreviation, e.g., “blood-brain barrier” is merged with “blood-brain-barrier” to become “BBB.” (4) Unification of plural forms of nouns into singular forms, e.g., “astrocytes” into “astrocyte.” (Supplementary file S1 lists the records of keyword merging and modification).

### 2.2 Visualization tools and analysis

**FIGURE 1:**
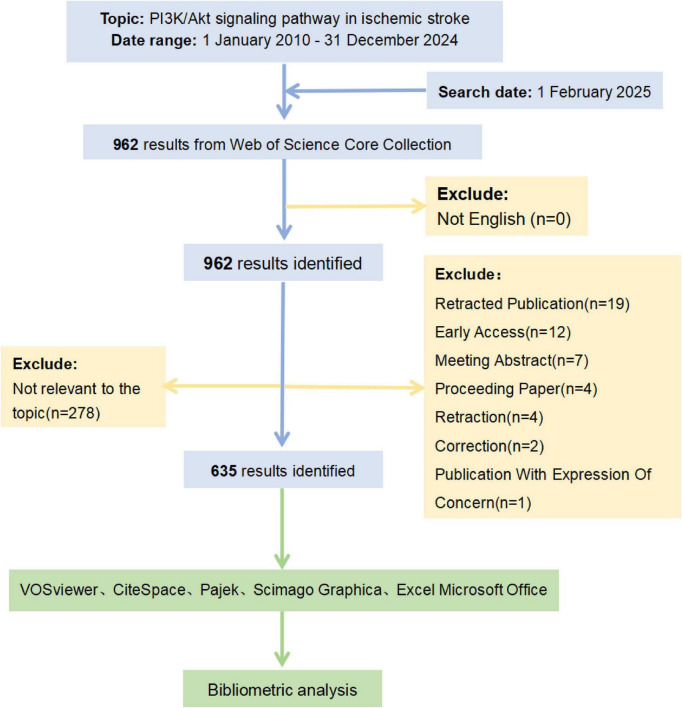
Flowchart for publication identification and analysis.

The main bibliometric analysis and data visualization tools used were VOSviewer software (version 1.6.19) and CiteSpace software (6.4.R1 advanced version). VOSviewer was used to construct a visual knowledge graph encompassing country, organization, author, co-citation author, journal, co-citation journal, and keyword co-occurrence network. Pajek (version 6.01) and Scimago Graphica (version 1.0.48) were used to refine clustering views for more intuitive visualization. CiteSpace primarily uses time-slicing techniques to construct a time series of network models, which evolve and integrate into a comprehensive overview network to analyze relevant literature systematically (Jiang et al., 2024). We used CiteSpace to generate a visual knowledge graph of journal biplot overlays and co-cited references. Meanwhile, we generated a cluster timeline by clustering the titles of the co-cited references. Additionally, we used CiteSpace to perform “burst detection” on keywords and references. To further characterize the data, we used Microsoft Office 2021 to analyze the exported data from VOSviewer and CiteSpace and construct a table of relevant analyses. A total of 635 articles on the PI3K/Akt in IS were analyzed, far exceeding the minimum recommended sample size of 200 for textual metrics analysis. Accordingly, the feasibility of this study was fully guaranteed. Figure 1 illustrates the flowchart for screening and analyzing the papers.

## 3 Results

### 3.1 Publication trends

**FIGURE 2:**
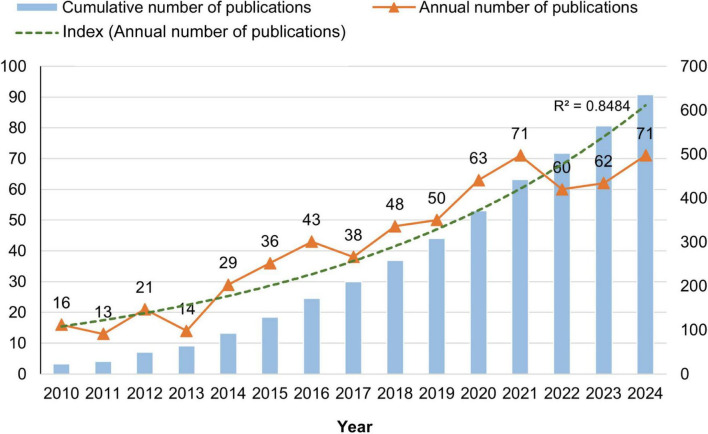
Trends in the annual volume of publications.

The search and screening of relevant literature from 2010 to 2024 yielded 635 publications, including 594 original articles (93.54%) and 41 reviews (6.46%). The number of publications was 16 in 2010, peaking at 71 in 2021. Although a slight decline occurred in 2022 (60 publications) and 2023 (62 publications), the number of publications rebounded to 71 in 2024, indicating sustained research interest. Figure 2 presents the annual publication trend of PI3K/Akt studies in IS. Overall, the number of published studies on PI3K/Akt in IS is increasing, with more relevant studies expected to be published in the future.

### 3.2 Analyze of countries and institutions

**FIGURE 3:**
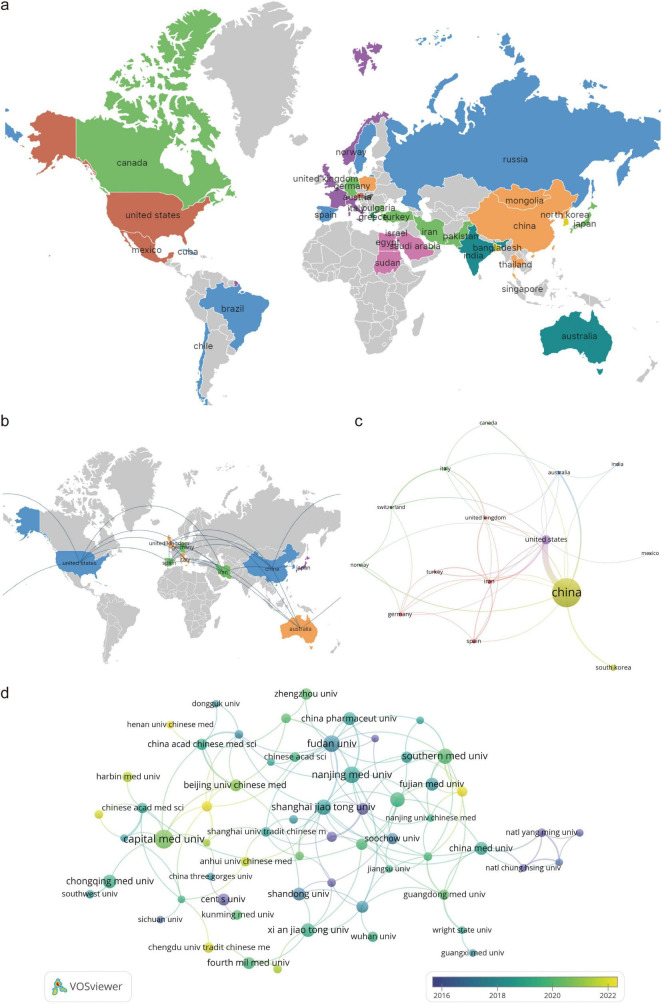
Visualization of countries and institutions involved in PI3K/Akt signaling pathway research in ischemic stroke (2010–2024); **(a)** geographic map of the global distribution of research results generated using VOSviewer with Scimago Graphica; **(b)** geographic map of global cooperation, generated using VOSviewer with Scimago Graphica, showing the cooperation between the 10 productive countries in this field; **(c)** cooperation network of different countries in the field generated using VOSviewer, where larger nodes represent more published table papers for the country and wider connecting lines between nodes indicate closer cooperation between countries; **(d)** timeline map of the cooperation network among different institutions generated using VOSviewer, where the closer the node color is to yellow, the later the year of collaboration.

Between 2010 and 2024, 38 countries published research on the PI3K/Akt in IS. The network of national collaborations was generated using VOSviewer, and Scimago Graphica was used to generate a map of the world’s geographical distribution of the research results. Figure 3a illustrates the distribution of countries contributing to this field on a global scale. Table 1 and Figure 3a present the top 10 most productive countries in this field, with China (533 papers), the United States (54 papers), and South Korea (26 papers) contributing the most. Although Japan published nine papers, none involved collaboration, suggesting that they need to be more actively involved in international collaboration to promote further development in this field. Citation analysis revealed that the United States has the highest average number of citations per paper (43.20), followed by the United Kingdom (39.17) and Australia (39.14) (Table 1). Figure 3c presents the collaboration among the top 10 countries in terms of published literature. Each node represents a country, with node size corresponding to publication volume and the thickness of the connecting lines reflecting the strength of collaboration. Figures 3b,c depict a wide range of cooperation among different countries, with China and the United States exhibiting the closest cooperation, indicating their crucial role in international cooperation.

**TABLE 1:** Top 10 most productive countries in PI3K/Akt signaling pathway research in ischemic stroke.

Rank	Countries	Publications	Citations	Average citations	Total link strength
1	China	533	14,941	28.03	46
2	United States	54	2,333	43.20	45
3	South Korea	26	807	31.04	3
4	Spain	12	427	35.58	7
5	Iran	11	384	34.91	15
6	Germany	10	171	17.10	5
7	Italy	9	313	34.78	5
8	Japan	9	205	22.78	0
9	Australia	7	274	39.14	9
10	United Kingdom	6	235	39.17	8

VOSviewer analysis identified 746 institutions contributing to the 635 papers. Table 2 lists the top 10 institutions in terms of the number of papers, all of which are based in China. Figure 3d depicts the network of institutional collaborations, revealing a dense network of collaborations primarily among Chinese institutions. These findings suggest that China plays a central role in PI3K/Akt research in IS. Strengthening collaborations with Chinese institutions could accelerate progress in this field. Figure 3d indicates that the widest distribution of links occurs in the yellow and green marked years, indicating that inter-institutional collaboration was most frequent between 2018 and 2020 and has continued beyond this period. Citation analysis of institutions (Table 2) reveals that Fudan University has the highest number of citations (557), followed by Capital Medical University (552), and Hebei Medical University (487).

**TABLE 2:** Top 10 most productive institutions in PI3K/Akt signaling pathway research in ischemic stroke.

Rank	Institutions	Publications	Citations	Average citations	Countries
1	Capital Medical University	19	552	29.05	China
2	Chongqing Medical University	12	388	32.33	China
3	Fudan University	16	557	34.81	China
4	Hebei Medical University	12	487	40.58	China
5	Nanjing Medical University	16	449	28.06	China
6	Shanghai Jiao Tong University	14	257	18.36	China
7	Southern Medical University	14	402	28.71	China
8	Xi’an Jiaotong University	12	203	16.92	China
9	Zhejiang Chinese Medical University	12	411	34.25	China
10	Zhejiang University	13	461	35.46	China

### 3.3 Analysis of authors and co-cited authors

**FIGURE 4:**
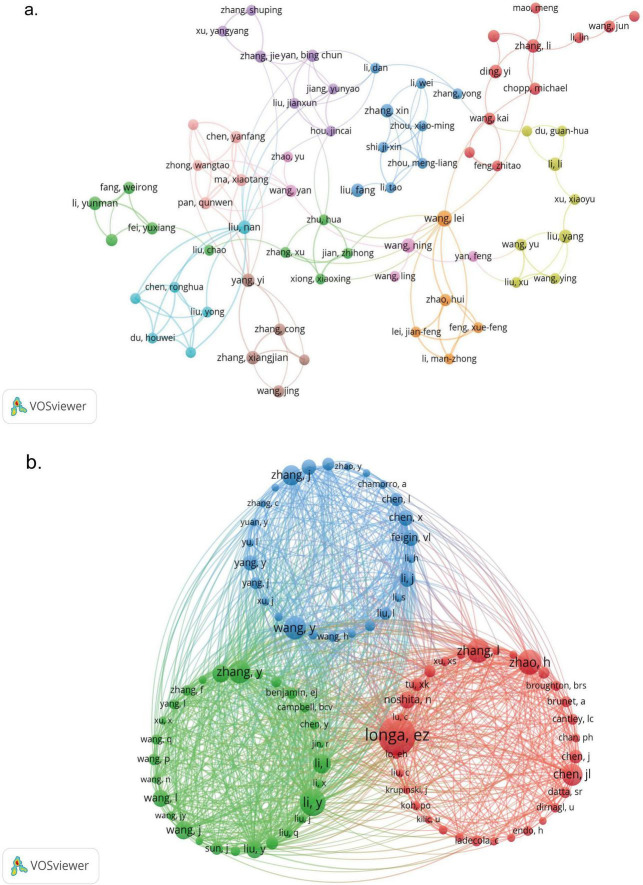
Visual network mapping of authors and co-cited authors in PI3K/Akt signaling pathway research in ischemic stroke; **(a)** network of authors with at least three publications generated using VOSviewer. Different nodes in the graph represent different core authors and different color bands indicate different collaborative clusters; **(b)** network of co-cited authors with at least 20 citations generated using VOSviewer and Pajek. Different nodes represent different co-cited authors, node size is proportional to the number of citations, and different color bands represent different clusters.

To construct a visual network map of authors and co-cited authors in PI3K/Akt research in IS, we used the VOSviewer visualization software and adjusted the clustering view using Pajek to make it more intuitive and clearer. The field comprises 3,885 authors. Table 3 lists the top 10 most prolific authors, with Wang Lei leading with 8 papers, followed by Wang Na and Wang Ning with 6 papers each. Chen Jing, Ding Yi, Li Li, Li Yunman, Liu Fang, Liu Li, and Liu Nan have each published five papers. Price’s law states that the minimum number of publications by core authors in a given field, m, is proportional to the square root of the number of publications by the most prolific authors, n_*max*_ which is given by m = 0.749 × ${\sqrt{n}}_{m\,a\,x}$ (Xu et al., 2024). Core authors in the field have at least 3 publications each (rounded upwards). Figure 4a illustrates the collaborative network of the 142 core authors, and it is evident that there are weak collaborative relationships between these core authors. This suggests that more cooperation and communication are required to expedite progress in the field. In terms of authors’ influence, Wang Lei has the highest number of citations at 389, while Liu Li has the highest average number of citations at 58 (Table 3), suggesting that their research findings have a significant international influence. Figure 4b demonstrates the network mapping of the co-cited authors, revealing three clusters. Among the 18,627 co-cited authors, 87 have been cited at least 20 times, with EZ Longa being the most cited (127 citations). Longa developed the middle cerebral artery occlusion model (MCAO) in 1989, a small-animal model of cerebral ischemia (Longa et al., 1989), which remains widely used worldwide.

**TABLE 3:** Top 10 most productive authors in PI3K/Akt signaling pathway research in ischemic stroke.

Rank	Authors	Publications	Citations	Average citations
1	Wang Lei	8	389	48.63
2	Wang Na	6	344	57.33
3	Wang Ning	6	328	54.67
4	Chen Jing	5	230	46.00
5	Ding Yi	5	71	14.20
6	Li Li	5	139	27.80
7	Li Yunman	5	97	19.40
8	Liu Fang	5	112	22.40
9	Liu Li	5	289	57.80
10	Liu Nan	5	84	16.80

### 3.4 Analysis of journals and co-cited journals

**FIGURE 5:**
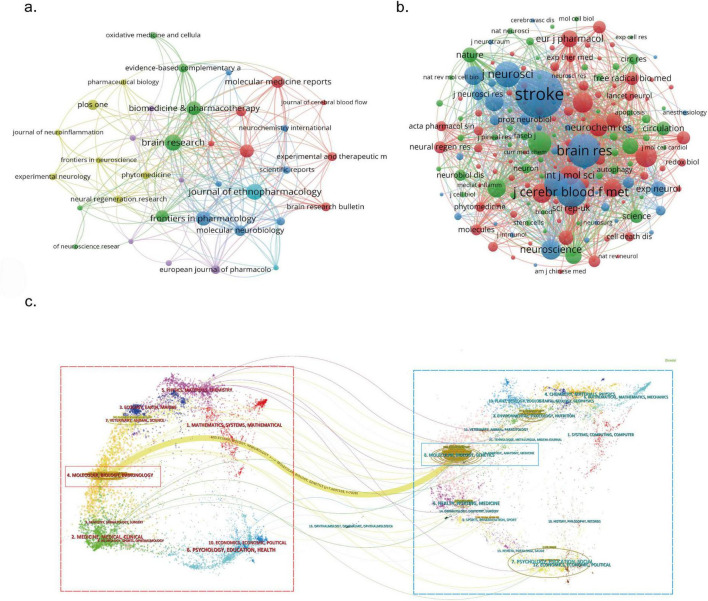
Visualized network mapping of relevant journals and co-cited journals in PI3K/Akt signaling pathway research in ischemic stroke; **(a)** network of journals that have published at least five papers generated using VOSviewer. Different nodes in the graph represent different journals, and the node size is proportional to the number of papers published in the journal; **(b)** network of co-cited journals with at least 40 citations generated using VOSviewer. Different nodes in the graph represent different co-cited journals, and the node size is proportional to the number of citations; **(c)** dual-map overlay of journals illustrating citation relationships between citing journals (knowledge frontier areas in red boxes) and cited journals (knowledge base areas in blue boxes). These citation links provide a visual representation of the interactions and connections between the topics of the two types of journals.

A total of 231 journals published papers on the PI3K/Akt in IS, with 36 journals publishing at least 5 papers (Figure 5a). Table 4 depicts the top 10 journals in terms of publications. The top 10 journals published 164 articles in the field, accounting for 25.83% of the total number of publications. *Brain Research* (22 papers), *Journal of Ethnopharmacology* (22 papers), and *Frontiers in Pharmacology* (19 papers) were the most prolific. Among the top 10 journals, the impact factor (IF) ranged from 2.70 to 6.90. Six journals, including the *Journal of Ethnopharmacology*, *Frontiers in Pharmacology*, *Biomedicine & Pharmacotherapy*, *Molecular Neurobiology*, *PLoS One*, and *International Journal of Molecular Sciences*, were ranked in the Q1 division based on the 2023 Journal Citation Reports (JCR). As illustrated in Figure 5b, 170 of the 3,243 co-cited journals have at least 40 citations, with different clusters represented by distinct colors. Table 5 details the top 10 most cited journals. Of these, *Stroke* tops the list with 7,925 citations and is also the journal with the highest IF (7.8 in 2023). *Neurorehabilitation and Neural Repair* (3,263 citations) and *Archives of Physical Medicine and Rehabilitation* (3,057 citations) follow closely.

**TABLE 4:** Top 10 most published journals in PI3K/Akt signaling pathway research in ischemic stroke.

Rank	Journals	Publications	Citations	2023 JCR (IF)
1	Brain Research	22	1,229	Q3 (2.7)
2	Journal of Ethnopharmacology	22	585	Q1 (4.8)
3	Frontiers in Pharmacology	19	437	Q1 (4.4)
4	Biomedicine & Pharmacotherapy	18	744	Q1 (6.9)
5	Neurochemical Research	17	661	Q2 (3.7)
6	Molecular Medicine Reports	15	385	Q2 (3.4)
7	Neuroscience	15	319	Q2 (2.9)
8	Molecular Neurobiology	13	361	Q1 (4.6)
9	PLoS One	12	390	Q1 (2.9)
10	International Journal of Molecular Sciences	11	244	Q1 (4.9)

**TABLE 5:** Top 10 most co-cited journals in PI3K/Akt signaling pathway research in ischemic stroke.

Rank	Co-cited journals	Citations	2023 JCR (IF)
1	Stroke	1,178	Q1 (7.8)
2	Brain Research	738	Q3 (2.7)
3	Journal of Cerebral Blood Flow and Metabolism	650	Q1 (4.9)
4	Journal of Neuroscience	525	Q1 (4.4)
5	PLoS One	467	Q1 (2.9)
6	Journal of Biological Chemistry	453	Q2 (4.0)
7	Journal of Ethnopharmacology	411	Q1 (4.8)
8	Neuroscience	374	Q2 (2.9)
9	Molecular Neurobiology	371	Q1 (4.6)
10	Frontiers in Pharmacology	362	Q1 (4.4)

Figure 5c depicts a dual-map overlay of journals. The red dashed box on the left represents citing journals (knowledge frontiers), and the blue dashed box on the right represents cited journals (foundational areas of knowledge). The interactive colored citation links present the strength of the association between the topics of these journals. The most prominent yellow citation path extends from “Molecular, Biology, and Genetics” (knowledge base) to “Molecular, Biology, and Immunology” (knowledge frontiers). This suggests that research on the PI3K/Akt in IS has focused on genetic regulation and immune response and has resulted in a relatively dense knowledge output. However, translational research, including target drug development and drug delivery, remains limited, indicating a gap between basic research findings and clinical applications in this field.

### 3.5 Analyzing cited papers and keywords

We employed VOSviewer software to conduct a citation analysis of the 635 papers included in the study. Our objective was to identify high-impact papers in the field of PI3K/Akt research in IS. This will facilitate our understanding of the research hotspots and trends in this field (Ravindran et al., 2019). Table 6 presents the top 10 cited papers in the field. Hou et al. (2018) published in *Genes & Diseases* [Q1 (6.9)], garnered the highest citation count, with 199 citations recorded. Its research focuses on the mechanisms of apoptosis in this field. Fu et al. (2022) published in the *Journal of Ethnopharmacology* [Q1 (4.8)], despite its late publication (2022), has received 162 citations and is in second place. This suggests that ferroptosis, a regulatory mechanism, is an emerging trend in the field of PI3K/Akt research in IS in recent years. Huang LF (Huang et al., 2018) published in the *Journal of Molecular Neuroscience* [Q3 (2.8)] was ranked third with 161 citations. Its study is indicative of the elevated level of interest and activity in autophagy research within this field. A significant finding was that six of the 10 highly cited papers concentrated on the active ingredients of traditional Chinese medicine (TCM) and the PI3K/AKT pathway, covering a span of 13 years (2010–2022) (Liu et al., 2010; Yao et al., 2012; Hou et al., 2018; Huang et al., 2018; Xie Y. et al., 2018; Fu et al., 2022). This finding suggests that this line of research not only continues to receive attention but also shows great potential for translation from basic research to clinical applications.

**TABLE 6:** Top 10 most cited papers in PI3K/Akt signaling pathway research in ischaemic stroke.

Rank	First author (year)	Papers	Citations	Journals	2023 JCR (IF)
1	(Hou et al., 2018)	Resveratrol provides neuroprotection by regulating the JAK2/STAT3/PI3K/AKT/mTOR pathway after stroke in rats	199	Genes & Diseases	Q1 (6.9)
2	(Fu et al., 2022)	Rehmannioside A improves cognitive impairment and alleviates ferroptosis via activating PI3K/AKT/Nrf2 and SLC7A11/GPX4 signaling pathway after ischemia	162	Journal of Ethnopharmacology	Q1 (4.8)
3	(Huang et al., 2018)	Neuroprotective effect of curcumin against cerebral ischemia-reperfusion via mediating autophagy and inflammation	161	Journal of Molecular Neuroscience	Q3 (2.8)
4	(Yao et al., 2012)	Quercetin attenuates cell apoptosis in focal cerebral ischemia rat brain via activation of BDNF–TrkB–PI3K/Akt signaling pathway	154	Neurochemical Research	Q2 (3.7)
5	(Zhu et al., 2013)	Both PI3K/Akt and ERK1/2 pathways participate in the protection by dexmedetomidine against transient focal cerebral ischemia/reperfusion injury in rats	153	Brain Research	Q3 (2.7)
6	(Xie Y. et al., 2018)	Protective effects and target network analysis of Ginsenoside Rg1 in cerebral ischemia and reperfusion injury: a comprehensive overview of experimental studies	141	Cells	Q2 (5.1)
7	(Liu et al., 2010)	Neuroprotection by baicalein in ischemic brain injury involves PTEN/AKT pathway	139	Journal of Neurochemistry	Q2 (4.2)
8	(Chen et al., 2013)	Neuroprotective effect of brain-derived neurotrophic factor mediated by autophagy through the PI3K/Akt/mTOR pathway	139	Molecular Medicine Reports	Q2 (3.4)
9	(Yuan et al., 2011)	Ischemic postconditioning protects brain from ischemia/reperfusion injury by attenuating endoplasmic reticulum stress-induced apoptosis through PI3K-Akt pathway	130	Brain Research	Q3 (2.7)
10	(Zhang et al., 2010)	Cerebrolysin enhances neurogenesis in the ischemic brain and improves functional outcome after stroke	124	Journal of Neuroscience Research	Q2 (2.9)

**FIGURE 6:**
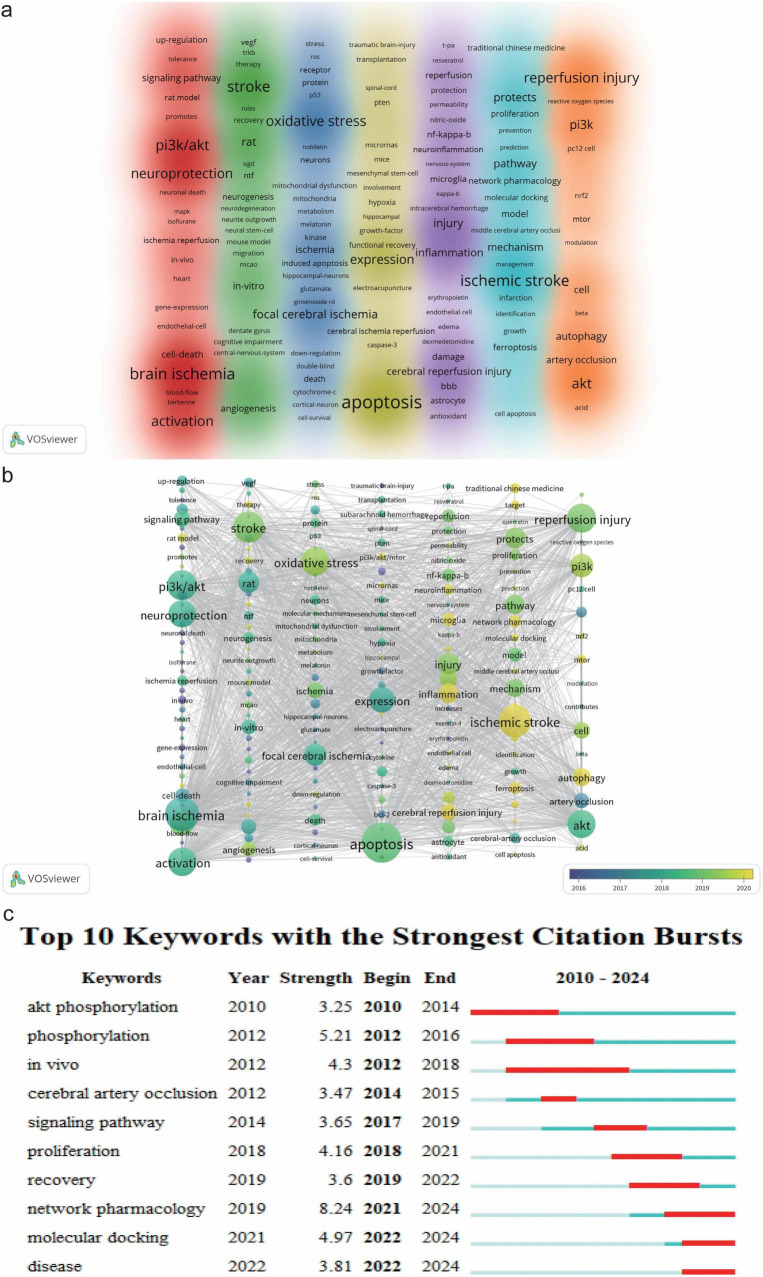
Visualization of keywords in PI3K/Akt signaling pathway research in ischemic stroke; **(a)** network mapping of keywords with a co-occurrence frequency of ≥ 5 generated by VOSviewer. Different colors in the graph represent different keyword clusters, and the darker the color and the more prominent the font, the higher the frequency of keywords; **(b)** temporal view of the keyword co-occurrence network generated using VOSviewer with Pajek. Larger nodes in the graph indicate a higher frequency of keyword occurrence, and the closer the node color is to yellow, the later the year of keyword occurrence; **(c)** Top 10 keywords in terms of burst strength mapped with the help of CiteSpace software. The higher the burst intensity of the keywords in the graph means that they have received more attention in a specific period. The start time reflects the duration of the keyword to maintain a high outburst status.

Keywords in scientific research papers reflect the topics of study. Analyzing keyword frequency and co-occurrence helps identify popular topics and emerging trends in a research field (van Eck and Waltman, 2010). We used VOSviewer to visualize keyword co-occurrences in PI3K/Akt research in IS and Pajek to adjust the clustering view. A total of 2,624 keywords were identified over the 15 years from 2010 to 2024, with 198 keywords co-occurring at least 5 times. Table 7 lists the top 10 most frequent keywords, including apoptosis (237), P13K/Akt (145), reperfusion injury (138), activation (135), oxidative stress (132), and neuroprotection (125). Figure 6a presents the co-occurrence network mapping of keywords in this domain. The keywords with strong relevance are grouped into seven colored clusters, all of which contain more than 15 keywords. The red cluster (41 keywords) is the largest and mainly focuses on PI3K/Akt activation to produce neuroprotection after cerebral ischemia/reperfusion injury, including terms brain ischemia, PI3K/Akt, activation and neuroprotection. The green keyword cluster (35 keywords) focuses on the neurorestorative effects of the PI3K/Akt pathway on stroke rats and *in-vitro* models. The keywords mainly include stroke, *in-vitro*, neurogenesis, angiogenesis, and rat. The blue cluster (30 keywords) focuses on the impact of oxidative stress and mitochondrial dysfunction on neuronal survival in this field, mainly involving the terms oxidative stress, focal cerebral ischemia, mitochondrial dysfunction, and cell-survival. The yellow cluster (27 keywords) focuses on neuronal apoptosis and expression in this field. The keywords cover apoptosis, expression, and caspase-3. The purple cluster (26 keywords) focuses on reperfusion injury and inflammation in this field and contains mainly the terms cerebral reperfusion injury, inflammation, and microglia. The cyan cluster (23 keywords) focuses on bioinformatics approaches to shed light on the protective mechanisms of the PI3K/Akt signaling pathway. The main terms involved are ischemic stroke, pathway, network pharmacology, and protects. The orange cluster (16 keywords) focuses mainly on autophagy mechanisms in reperfusion injury in this field. Terms such as autophagy, PI3k, Akt, mTOR. These results indicate research hotspots in this field.

**TABLE 7:** Top 10 most frequent keywords in PI3K/Akt signaling pathway research in ischemic stroke.

Rank	Keywords	Occurrences	Total link strength
1	Apoptosis	237	1843
2	Brain ischemia	182	1431
3	Ischemic stroke	167	1077
4	Stroke	155	1157
5	PI3k/Akt	145	1126
6	Reperfusion injury	138	1106
7	Activation	135	1103
8	Akt	133	1134
9	Oxidative stress	132	987
10	Neuroprotection	125	1016

The temporal mapping of keyword networks was generated using VOSviewer based on the average year of first keyword occurrence, and the clustering layout was optimally adjusted using Pajek (Figure 6b). The figure depicts that since 2020, ischemic stroke, cerebral reperfusion injury, autophagy, inflammation, microglia, ferroptosis, network pharmacology, and traditional Chinese medicine (TCM) have been the frequently appearing keywords. This suggests that autophagy and neuroinflammation are not only current research hotspots but also future trends in PI3K/Akt studies in IS. Notably, the role of ferroptosis in this field is gradually expanding, indicating the potential for more relevant studies. Additionally, targeting the PI3K/Akt with TCM for IS treatment is expected to become a key direction for translating basic research findings into clinical applications. We generated the top 10 keywords in terms of burst strength using CiteSpace. Figure 6c depicts that keywords such as cyber pharmacology (burst strength of 8.24; 2021–2024) and molecular docking (burst strength of 4.97; 2022–2024) exhibited strong burst trends, which will probably continue in the future. This indicates that using bioinformatics approaches to investigate the targeting of PI3K/Akt by active ingredients of TCM and their application in IS treatment has become an important direction for future research in this field.

### 3.6 Analysis of co-cited references

**FIGURE 7:**
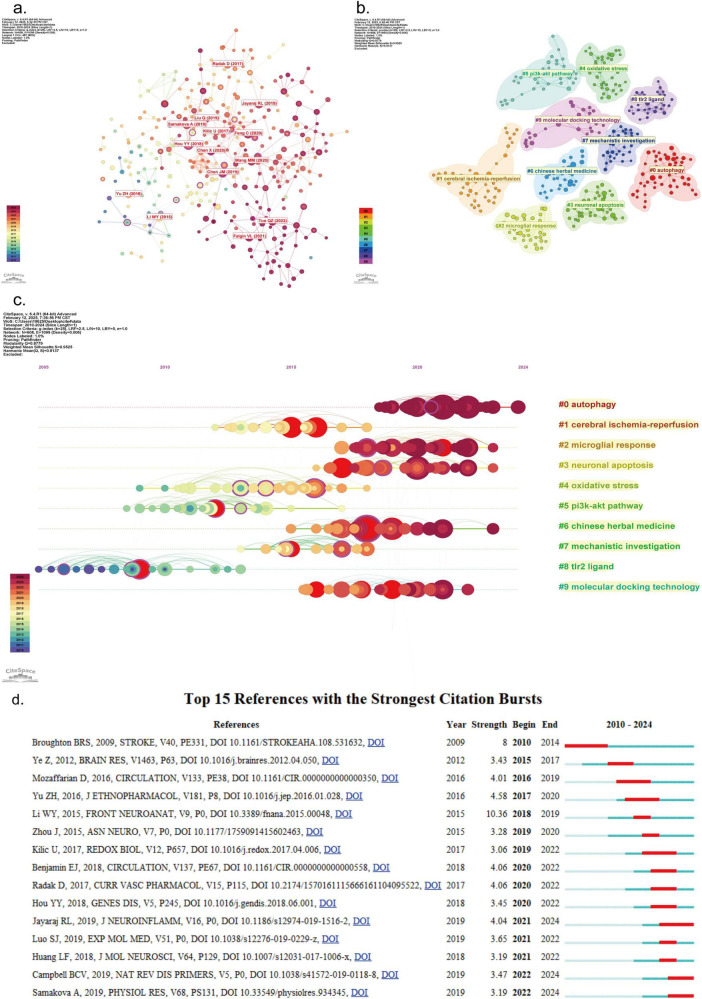
Visualization of co-cited references in PI3K/Akt signaling pathway research in ischemic stroke; **(a)** co-citation network of references mapped using CiteSpace software. The size of the nodes in the graph is proportional to the frequency of co-citation, while the connecting lines between the nodes indicate the correlation between different studies, with more connecting lines indicating higher centrality. **(b)** Distribution of the top 10 major clusters of co-cited references mapped using CiteSpace software; **(c)** timeline mapping of the top 10 major clusters of co-cited references plotted using CiteSpace software. The map illustrates the temporal progression of the clusters through horizontal arrangement, with nodes within the same cluster ordered from left (early) to right (recent) based on their respective publication times. The size of the nodes serves as an intuitive metric for assessing the popularity of literature during different periods. **(d)** Top 15 references with the strongest citation bursts. plotted using CiteSpace software. As illustrated in the graph, burst intensity is indicative of the amount of attention a reference has received during a specified period, while onset time signifies the duration of the elevated burst state.

Analysis of co-cited references helps to identify the knowledge base and influential articles within a large body of literature, enabling a comprehensive exploration of the field (Zhu et al., 2023). In total, 26,555 references were cited across 635 articles on the PI3K/Akt in IS. Using CiteSpace software, we mapped the co-citation network of these references (Figure 7a). Table 8 details the top 10 co-cited references based on high frequency and centrality, comprising 7 original articles and 3 reviews. It is noteworthy that Hou et al. (2018) attained the top position in terms of co-citation frequency (15) and centrality (0.27). Chen et al. (2019) was the runner-up, with a centrality of 0.25. These studies represent the core nodes in Figure 7a, which establish connections between diverse research directions and provide significant theoretical support and a bridging role for subsequent studies. Feng et al. (2020) exhibited the second-highest co-citation frequency (14), though its centrality (0.01) was minimal. In a manner analogous to Hou et al. (2018), Feng et al. (2020) concentrated on the neuroprotective effects of TCM by targeting the PI3K/Akt pathway. Chen et al. (2020) (centrality 0.11, ranked third) employed a research strategy that integrated proteomics analysis techniques with TCM.

**TABLE 8:** Top 10 co-cited references based on high frequency and centrality in PI3K/Akt signaling pathway research in ischemic stroke.

Rank	First author	Count	Centrality	Year	Cited reference	Journal	2023 JCR (IF)
1	(Hou et al., 2018)	15	0.27	2018	Resveratrol provides neuroprotection by regulating the JAK2/STAT3/PI3K/AKT/mTOR pathway after stroke in rats	Genes & Diseases	Q1 (6.9)
2	(Feng et al., 2020)	14	0.02	2020	Neuroprotective effect of Danhong injection on cerebral ischemia-reperfusion injury in rats by activation of the PI3K/Akt pathway	Frontiers in Pharmacology	Q1 (4.4)
3	(Feigin et al., 2021)	14	0.01	2021	Global, regional, and national burden of stroke and its risk factors, 1990–2019: a systematic analysis for the global burden of disease study 2019	Lancet Neurology	Q1 (46.5)
4	(Chen et al., 2019)	13	0.25	2019	Ginsenoside Rg1 promotes cerebral angiogenesis via the PI3K/Akt/mTOR signaling pathway in ischemic mice	European Journal of Pharmacology	Q1 (4.2)
5	(Jayaraj et al., 2019)	13	0.01	2019	Neuroinflammation: friend and foe for ischemic stroke	Journal of Neuroinflammation	Q1 (9.3)
6	(Tuo et al., 2022)	12	0.03	2022	Mechanisms of neuronal cell death in ischemic stroke and their therapeutic implications	Medicinal Research Reviews	Q1 (10.9)
7	(Samakova et al., 2019)	11	0.07	2019	The PI3k/Akt pathway is associated with angiogenesis, oxidative stress and survival of mesenchymal stem cells in pathophysiologic condition in ischemia	Physiological Research	Q3 (1.9)
8	(Chen et al., 2020)	10	0.11	2020	Proteomics-guided study on Buyang Huanwu decoction for its neuroprotective and neurogenic mechanisms for transient ischemic stroke: involvements of EGFR/PI3K/Akt/Bad/14-3-3 and Jak2/Stat3/Cyclin D1 signaling cascades	Molecular Neurobiology	Q1 (4.6)
9	(Kilic et al., 2017)	10	0.06	2017	Particular phosphorylation of PI3K/Akt on Thr308 via PDK-1 and PTEN mediates melatonin’s neuroprotective activity after focal cerebral ischemia in mice	Redox Biology	Q1 (10.7)
10	(Li et al., 2015)	10	0.05	2015	Neuroprotective effects of DAHP and triptolide in focal cerebral ischemia via apoptosis inhibition and PI3K/Akt/mTOR pathway activation	Frontiers in Neuroanatomy	Q1 (2.1)

To further investigate research hotspots, we used CiteSpace software to generate 22 clusters. The modularity value and weighted average profile value of the clusters were 0.8779 and 0.9137, respectively, indicating high clustering confidence. Figure 7b presents the distribution of the top 10 clusters. Similarly, in Figure 7c, the horizontal position of the nodes on the time axis represents when the references were cited, while node size represents the frequency of citation. Recent research hotspots in the PI3K/Akt in IS include cluster 0# (autophagy), cluster 2# (microglial response), cluster 3# (neuronal apoptosis), cluster 6# (Chinese herbal medicine), and cluster 9# (molecular docking technology).

A citation burst indicates a significant increase in attention to a specific article in a specific research area within a certain time frame (Xu et al., 2024). References with high values in the strength column are usually milestones in scientific cartographic research (Chen, 2004). Figure 7d presents the top 15 references with the strongest citation bursts. The earliest and longest-spanning reference is “ DOI 10.1161/STROKEAHA.108.53163” (Broughton et al., 2009), with a citation burst concentrated between 2010 and 2014, focusing on apoptosis after stroke. The reference with the highest citation burst strength (10.36) is “ DOI 10.3389/fnana.2015.0004” (Li et al., 2015), which focuses on neuroapoptosis by targeting the PI3K/Akt to provide neuroprotection against stroke. Notably, the reference “ DOI 10.1186/s12974-019-1516-2” maintained a citation peak until 2024. It focuses on post-acute stroke neurological inflammatory mechanisms (Jayaraj et al., 2019). This suggests that inflammation is a current research focus in PI3K/Akt in IS and indicates future research directions.

## 4 Discussion

### 4.1 Summary of basic information

This study used bibliometrics to analyze 635 original articles and reviews on PI3K/Akt in IS from 2010 to 2024. The number of publications increased steadily each year, peaking in 2021 with 71 publications. Among the 38 countries analyzed, China ranked first with 533 publications, followed by the United States and South Korea. China and the United States exhibited the closest collaboration and made significant contributions to the field. The top 10 research institutions, including Capital Medical University, Chongqing Medical University, and Fudan University, are all based in China, and a relatively close network of cooperation has been established between these institutions. However, further international institutional collaboration is needed to advance the field. Analysis of authors and co-cited authors identified 3,885 authors and 18,627 co-cited authors. Wang Lei was the most prolific author, publishing 8 papers, while Liu Li had the highest average number of citations at 57.80. Among the co-cited authors, EZ Longa had the highest number of citations at 127. This fully reflects their influence and Contribution To The Field.

The 635 articles analyzed were from 231 journals. The top three journals in terms of publication volume were *Brain Research*, *Journal of Ethnopharmacology*, and *Frontiers in Pharmacology*, collectively accounting for 25.83% of all published articles, indicating their significant contributions to the field. *Stroke* was the most frequently cited journal, with 7,925 citations, highlighting its authority in PI3K/Akt research in IS. However, the dual-map overlay of journals revealed a gap between basic research findings and clinical applications.

### 4.2 Research hotspots

We identified several major hotspots for PI3K/Akt research in IS by visualizing and analyzing highly cited papers, keywords, and co-cited literature. These hotspots mainly focus on the pathological mechanisms and targeted therapies of IS.

#### 4.2.1 Apoptosis

Apoptosis is an orderly and efficient process of programmed cell death under normal physiological conditions (Fleisher, 1997). It is responsible for removing damaged cells, and it is regulated by key apoptotic proteins (Elmore, 2007). However, in IS, neuroapoptosis occurs excessively (Elmore, 2007). Activation of the PI3K/Akt inhibits apoptosis in neuronal cells by upregulating the expression of anti-apoptotic proteins (e.g., Bcl-2) and downregulating pro-apoptotic proteins (e.g., Bax, caspase-3, and caspase-9) following PI3K/Akt phosphorylation, thereby inhibiting the apoptotic cascade (Yu et al., 2016; Xu et al., 2021). Excessive apoptosis of neuronal cells is a hallmark of ischemic/reperfusion (I/R) injury in IS. The PI3K/Akt, activated by miR-18b, reduced infarct size, attenuated neurological deficits, and inhibited apoptosis in a mouse MCAO model and an *in-vitro* oxygen-glucose deprivation/reperfusion model to prevent cerebral I/R injury (Min et al., 2020). However, a recent study published in *Redox Biology* reported that after MCAO induction, PI3K/Akt activation promoted astrocyte proliferation and the release of pro-inflammatory cytokines, including interleukin-1 beta (IL-1β), IL-6, and tumor necrosis factor-alpha (TNF-α), which exacerbated apoptosis and resulted in brain tissue damage (Cai et al., 2023). This seemingly contradictory finding may arise from the dual role of astrocytes, the most abundant glial cells in the mammalian brain, in IS recovery (Cai et al., 2023). Thus, PI3K/Akt activation after IS onset is not always beneficial to neuronal cells, and the different mechanisms involved in different cells can have different effects on the pathological mechanisms of IS. Further studies are needed to investigate this.

#### 4.2.2 Autophagy

Autophagy is a crucial process that helps cells maintain intracellular homeostasis by degrading and recycling proteins and organelles (Liu S. et al., 2023). Autophagy has dual functions and typically protects cells. In IS, pathological factors, including oxidative stress, mitochondrial dysfunction, and endoplasmic reticulum stress, can dysregulate autophagic processes and over-activate autophagic fluxes, leading to necrotic and apoptotic cascade responses (Wang et al., 2018). The P13K/Akt is closely linked to autophagy in IS; however, its ability to regulate autophagy is typically dependent on the activation of downstream mTOR (a protein that maintains cellular homeostasis) (Perez-Alvarez et al., 2018). Autophagy-related proteins [for example, Beclin-1 and light chain 3 (LC3)] and apoptosis-related proteins are significantly overexpressed during cerebral I/R injury, while the anti-apoptotic protein is suppressed. However, activating the PI3K/Akt/mTOR can inhibit apoptosis and excessive autophagy induced by brain I/R injury, thereby protecting the neurons (He et al., 2019; Meng et al., 2021). In a study, the PI3K/Akt mitigated cerebral IS by moderately activating the autophagy mechanism, resulting in a neuroprotective effect (Zhao et al., 2023). This suggests that the role of autophagy in IS remains controversial and that its effects on neurorecovery are not entirely negative. This controversy may explain why autophagy has recently become a research hotspot in studies on the PI3K/Akt in IS.

#### 4.2.3 Microglia and neuroinflammation

Microglia are resident innate immune cells in the brain that continuously monitor the brain’s microenvironment and respond to changes to maintain normal brain function. They are among the first cells to respond when IS occurs (Doyle et al., 2008). Microglia-mediated neuroinflammation is a significant cause of secondary brain injury during I/R. During this process, microglia differentiate into two distinct phenotypes depending on the surrounding environment: M1, which releases pro-inflammatory factors, such as CD86, iNOS(an oxidative stress molecule), TNF-α, that exacerbate tissue damage, and M2, which releases anti-inflammatory factors, such as CD206, IL-10, and transforming growth factor beta, that promote neural repair (Murray et al., 2014; Li et al., 2023; Ri et al., 2023). The PI3K/Akt is considered a core pathway that suppresses the M1 phenotype and enhances the M2 phenotype (Ri et al., 2023). Activating the PI3K/Akt and its downstream proteins, such as nuclear factor-kappaB (NF-κB) and mTOR, can induce microglia polarization toward the M2 type, inhibit apoptosis, attenuate oxidative stress and neuroinflammation, and exert neuroprotective effects in IS (Yan et al., 2022; Duan et al., 2024). Modulating microglia polarization from M1 to M2 to inhibit neuroinflammation is a promising strategy to achieve neuroprotection and promote neurological recovery after IS.

#### 4.2.4 Oxidative stress

Sies (2015) first introduced the concept of oxidative stress in 1985, and it has been widely used in biomedical applications. Many neurological diseases, especially IS, are closely linked to oxidative stress (Zheng et al., 2013). A hallmark of oxidative stress following IS is the imbalance between excessive production and untimely clearance of reactive oxygen species (Cai et al., 2023). Oxidative stress contributes to multiple pathomechanistic IS processes. Oxidative stress may directly regulate and promote neuronal apoptotic processes after IS before mitochondrial dysfunction triggers the release of pro-apoptotic proteins (Niizuma et al., 2009). However, activation of the P13K/Akt signaling pathway significantly reduces apoptosis triggered by oxidative stress and promotes angiogenesis in the rat model, thereby reducing brain tissue damage and accelerating neurological recovery (Yang F. et al., 2022; He et al., 2024; Zhang X. et al., 2024). In addition, oxidative stress activates NF-κB, which promotes the expression of inflammatory factors, exacerbating nerve damage. Upregulating PI3K and Akt can effectively inhibit this process (Xu et al., 2023). Although Figure 7 demonstrates that oxidative stress as a single theme is no longer a research hotspot, the regulation of oxidative stress-mediated apoptosis and inflammatory responses in neuronal cells via the PI3K/Akt remains a hotspot in IS research.

#### 4.2.5 Bioinformatics approaches and traditional Chinese medicine

Bioinformatics is becoming increasingly important in medicine and translational drug discovery and development (Wooller et al., 2017). Histological analysis, network pharmacology analysis, and molecular docking are some of the most commonly used bioinformatics approaches. Biological networks are first constructed and analyzed using network pharmacology to identify core drug targets to treat diseases. Subsequently, histological analyses are used to elucidate the mechanism of action of the drug and assess the suitability of the target. Finally, molecular docking is used to validate these findings (Wang et al., 2023; Yuan et al., 2023). Recently, these analytical methods have been increasingly applied to the study of TCM for IS treatment. The PI3K/Akt has been repeatedly demonstrated to be an important target in the study of IS.

Several TCM compound formulations and single components stand out, including Buyang Huanwu Decoction (Chen et al., 2020), Yiqi Tongluo Granule (Yuan et al., 2023), Honghua class injections (Feng et al., 2020; Zhou et al., 2021), Scutellarin (Duan et al., 2024), and Salvianolic Acid C (Yang F. et al., 2022). It is worth mentioning that Honghua class injections are significantly effective in treating acute IS in 120 randomized controlled trials (RCT), which involved a total of 12,658 patients (Li et al., 2022). These results suggest that TCM’s basic research targeting PI3K/Akt for IS treatment is gradually being translated into clinical applications. Additionally, bioinformatics technology will become an indispensable tool for TCM research. It is also a key technology that will drive clinical translation in this field. Studies exploring the complex regulatory mechanisms of TCM for IS have revealed key targets, such as the PI3K/Akt pathway. These findings strongly demonstrate the scientific validity of TCM’s efficacy.

### 4.3 Research trends

By analyzing keyword co-occurrence and reference clustering, the trend of PI3K/Akt pathway research in IS was identified. Autophagy, neuroinflammation, and microglia are all current research hotspots and crucial directions for future research. A close and complex relationship has been observed between oxidative stress, apoptosis, and neuroinflammation. Consequently, subsequent investigations on PI3K/Akt in IS may facilitate a more profound examination of the mechanisms through which oxidative stress instigates apoptosis and neuroinflammation. Notably, ferroptosis (iron-dependent programmed apoptosis) is gaining attention in IS research related to the PI3K/Akt. Inhibiting ferroptosis in IS reduces cellular autophagy, promotes microglia polarization toward the M2 phenotype, alleviates neurological deficits, and improves cognition. These mechanisms are closely linked to PI3K/Akt pathway activation (Fu et al., 2022; Zhao et al., 2023; Zhang Y. et al., 2024). As a result, further studies on the role of ferroptosis in IS pathogenesis and its regulation via the PI3K/Akt could provide insights into therapeutic strategies against IS. Additionally, targeting the PI3K/Akt for IS treatment has been extensively supported by bioinformatics analyses, reinforcing the rationale for using TCM. Consequently, this direction is expected to become an important trend in translating basic research findings into clinical applications.

**FIGURE 8:**
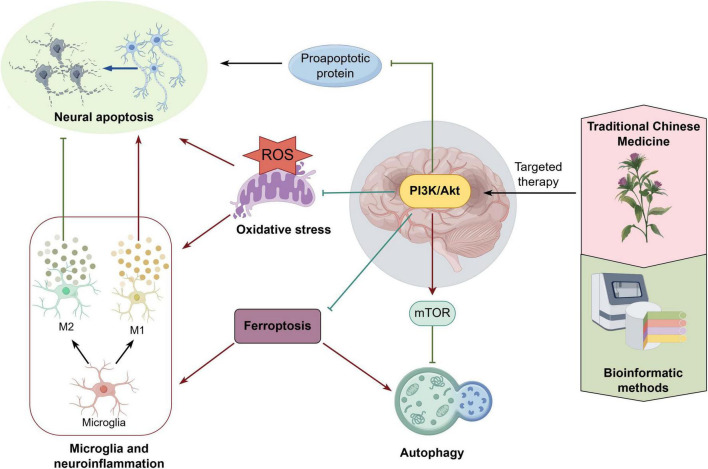
This figure illustrates the dynamic interrelationships among the various key regulatory mechanisms involved in the activation of the PI3K/Akt signaling pathway and their intervention strategies in IS. It encompasses current research topics of significant interest and pioneering directions in the field. Specifically, the activation of PI3K/Akt effectively inhibits oxidative stress and reduces the expression of pro-apoptotic proteins, thereby reducing neuronal apoptosis. It has been demonstrated to impede microglia M1-type polarization induced by oxidative stress, thereby attenuating neuroinflammatory injury. PI3K/Akt exerts neuroprotective effects through the regulation of downstream mTOR proteins and the inhibition of the process of autophagy. Of particular significance is the observation that the activation of PI3K/Akt has been demonstrated to inhibit ferroptosis in IS, reduce cellular autophagy, and promote microglia polarization toward an M2-type phenotype. This, in turn, has been shown to exert anti-inflammatory and repair effects. The present study explores the significance of TCM in the treatment of IS, with a particular focus on its targeted engagement with the PI3K/Akt pathway. Bioinformatics offers a scientific foundation for the efficacy of TCM, underscoring its potential as a therapeutic modality. By Figdraw.

Through an in-depth analysis of the salient topics and emergent trends in this domain, we offer a concise depiction in Figure 8 of the dynamic correlation between the research focal points and the emerging regulatory mechanisms of PI3K/Akt in IS. This contributes to an in-depth understanding of the pathomechanisms of IS and provides important guidance for the development of targeted therapeutic strategies.

### 4.4 Limitations and improvement strategies

It is imperative to acknowledge the limitations inherent in this study. During the literature screening process, the study’s scope was constrained to the WoSCC database. This decision was influenced by the recognition that prevailing software, such as VOSviewer and CiteSpace, encounter difficulties in accommodating data standardization and compatibility issues when conducting cross-database analysis. Nevertheless, this methodological approach may potentially result in the exclusion of significant research findings within this field. Concurrently, the most recent research results from January 1, 2025, to the retrieval date were not incorporated into the analysis, which could have limited the comprehensive grasp of the cutting-edge developments in IS-related PI3K/Akt research. At the technical analysis level, CiteSpace software was limited in authorship identification flaws, including its inability to distinguish between first and corresponding authors and differentiate between authors with the same name from different institutions. Furthermore, its clustering analysis was susceptible to data loss and result bias owing to the lack of parameter standardization.

These limitations may be overcome in the future through the implementation of artificial intelligence technologies. To minimize the probability of overlooking pertinent literature, it is imperative to devise intelligent cross-library standardization instruments that seamlessly integrate disparate data formats from numerous sources. When employed in conjunction with ORCID and other unique identifiers, it establishes an accurate author identification system and effectively addresses the ambiguity problem posed by scholars with identical names. The utilization of machine learning algorithms facilitates the dynamic optimization of data cleaning and parameter standardization processes. This approach is designed to mitigate the impact of data noise and systematic bias on cluster analysis. These measures are expected to enhance the depth and reliability of bibliometric results on a comprehensive scale.

## 5 Conclusion and prospects

For this study, we performed an objective, in-depth bibliometric analysis of high-quality literature on PI3K/Akt from the WoSCC database. We used a synergistic analysis approach with VOSviewer and CiteSpace. Using this approach, we gained a comprehensive understanding of the evolution of research in this field over the past fifteen years. We also identified current research hotspots, including pathological mechanisms such as apoptosis, autophagy, inflammation, and oxidative stress. Additionally, we identified an emerging regulatory mechanism in this field: ferroptosis. Our study reveals that combining bioinformatics technology and TCM in PI3K/Akt research for IS is key to translating basic results into clinical applications in the future. However, most RCT studies lack adequate monitoring of the adverse effects of TCM during clinical translation (Li et al., 2022). The safety issues of TCM, such as unknown toxicity, drug-drug interactions, and the risks of long-term use, need to be thoroughly investigated. Meanwhile, the bioavailability of natural active ingredients in TCM is often challenged by factors such as a strong first-pass effect and difficulty penetrating the blood-brain barrier (BBB). Additionally, there are regulatory challenges, including standardizing the quality of TCM and defining active ingredients. It is important to note that these challenges could impede the future clinical translation of PI3K/Akt research in IS. The quality control and clinical monitoring of TCM must be improved in the future to ensure safety. At the same time, advanced technologies, such as nanocarriers and structural modifications, should be used to increase bioavailability and overcome the BBB (Zhao et al., 2022). In the future, it is crucial to construct an evidence-based evaluation system that considers the complex regulatory features of TCM and meets the requirements of international drug regulatory agencies (e.g., the FDA and EMA) to promote the translation process from basic research to clinical application in the field of PI3K/Akt research in IS.

## Data Availability

The original contributions presented in the study are included in the article/Supplementary material, further inquiries can be directed to the corresponding author/s.
